# The Relationship Between Strap Use and Classification Score in Elite Wheelchair Basketball Players

**DOI:** 10.3390/sports13070222

**Published:** 2025-07-08

**Authors:** Giacomo Farì, Francesco Quarta, Sara Clelia Longo, Fernando Zappile, Laura Masiero, Giustino Varrassi, Andrea Bernetti

**Affiliations:** 1 Department of Experimental Medicine (Di.Me.S.), University of Salento, 73100 Lecce, Italy; andrea.bernetti@unisalento.it; 2 Italian Wheelchair Basketball Federation (Federazione Italiana Pallacanestro in Carrozzina, FIPIC), 00188 Rome, Italy; hassia@tiscali.it; 3 Department of Biological and Environmental Science and Technologies (Di.S.Te.B.A.), University of Salento, 73100 Lecce, Italy; 4 Rehabilitation Unit, M. Paternò Arezzo Hospital, Provincial Health Authority, 97100 Ragusa, Italy; clelialongo29@gmail.com; 5 Department of Statistical Science, University of Padova, 35121 Padova, Italy; laura.masiero.3@gmail.com; 6 Department of Pain Medicine, Paolo Procacci Foundation, 00193 Rome, Italy; g.varrassi@fondazioneprocacci.org; 7 Infradepartmental University Program of Physical and Rehabilitation Medicine, “V. Fazzi” Hospital, ASL Lecce, 73100 Lecce, Italy

**Keywords:** wheelchair basketball, Paralympics, classification, sport, injury, disability

## Abstract

Wheelchair basketball (WB) grants important benefits for people with disabilities but also presents a relevant risk of injury. Wheelchair straps are restraint devices that can improve safety and performance, but limited research has explored their use in WB. This study aims to analyze the use of different types of straps among professional WB players, according to classification score. A cross-sectional study was conducted through an online survey. Participants were divided into two groups based on classification score: low-point players (LPPs; 1.0–2.5), who have greater physical impairment, and high-point players (HPPs; 3.0–4.5), who have lower physical impairment. A total of 82 WB players participated (43 LPPs; 39 HPPs). The Chi-squared test was used to compare variables between groups. Significant differences emerged: chest (*p* = 0.036), abdominal (*p* = 0.036), and foot (*p* = 0.016) straps were more frequently used by LPPs, while thigh (*p* = 0.020) and leg (*p* = 0.050) straps were more common among HPPs. No significant difference was found for pelvic strap. Straps used in WB vary with classification score, reflecting the influence of functional ability. These findings offer insights into individualized wheelchair setup and classification procedures. Further studies are needed to expand knowledge on this topic.

## 1. Introduction

Wheelchair basketball (WB) is a widely recognized and increasingly popular Paralympic sport worldwide [1,2]. WB offers numerous physical, psychological, and social benefits for people with disabilities [3,4,5]. The rules of this sport are largely similar to those of stand-up basketball, with specific adaptations related to the use of wheelchairs and the need to reduce the impact of players’ physical impairments [6]. In fact, athletes with a variety of impairments, and consequently with various levels of residual functions, participate in WB.

In order to minimize the effect of these differences and to make the game as fair as possible, each athlete is assigned a classification score ranging from 1.0 to 4.5 with half-point increments. This results in a total of eight classes (1.0, 1.5, 2.0, 2.5, 3.0, 3.5, 4.0, 4.5), where lower scores correspond to greater levels of physical impairment. In this regard, the total amount of the classification scores of the five players on the court for each team must not exceed 14.0 at any given time [6]. Classification considers several functional aspects, and one of the most important is the player’s volume of action. According to the International Wheelchair Basketball Federation (IWBF), the volume of action refers to the space a player can actively reach by moving in any direction and then returning to an upright seated position without using the wheelchair or their upper limbs for support [7]. In other words, it represents the maximum functional range of movement across all three planes of motion (sagittal, frontal, and transverse). Players with higher classification scores demonstrate greater trunk control across all planes of movement, resulting in an increased volume of actions compared to players with lower classification scores [7].

While functional classification ensures fair competition, the specific demands and biomechanics of WB expose all players to a significant risk of injury due to the repetitive high-intensity actions and frequent contact situations [8,9,10,11]. Movements such as wheelchair propulsion and overhead actions like passing or shooting place continuous stress on the upper limbs, particularly the shoulder region, contributing to overload and repeated microtrauma [12,13,14]. Additionally, as a contact sport, WB involves frequent collisions and falls, which are associated with longer recovery periods compared to overuse injuries [15]. Given this risk profile, implementing effective injury prevention strategies is crucial. Specifically, falls in WB have been increasingly reported in recent years, and the risk of falling in this sport is significantly higher compared to other wheelchair sports [16]. Specifically, fractures, lacerations, and contusions are the most common consequences [17]. Moreover, in WB, both the frequency and the characteristics of falls are influenced by the players’ classification score, with a higher incidence observed among players in the higher classes [18].

In this context, the use of straps represents a practical and effective injury prevention strategy, particularly for minimizing the consequences related to wheelchair falls [19,20,21]. Straps or belts are restraint devices that can be fastened to various components of the wheelchair, securing different parts of the body, such as the chest, trunk, pelvis, thighs, legs, and feet. These systems contribute to enhancing players’ balance and optimizing seating position within the wheelchair [19,22], particularly during physical contact situations, which are typical in WB. Furthermore, the stabilizing function of straps is especially relevant in dynamic game situations where both balance and trunk control are critical.

As reported by the IWBF rules, there are no limitations to securing a player in a wheelchair. In fact, players are free to add or remove strapping as needed without it affecting their classification or requiring documentation on the player’s card. However, a limitation is related to players who have double leg amputations. They may secure their legs below the knees only if specified on their player classification card, while strapping above the knees or the legs to each other is permitted without affecting classification. Moreover, for players with a below-knee amputation, the use of straps must be noted on the player’s classification card, as this condition is considered equivalent to a complete leg and confers a significant functional advantage [23]. Nevertheless, it appears that the pursuit of excessive stability through the use of straps may compromise the mobility of wheelchair athletes by restricting their movement [24]. Despite this, straps have been shown to improve performance in specific WB tests involving sprinting and wheelchair maneuverability, both with and without the ball [25].

Although some studies have addressed equipment configuration and restraint devices in WB [19,20,21,22,24,25], the existing scientific literature lacks studies specifically focused on the use of straps in WB in relation to players’ classification scores. Previous research has explored wheelchair setup without accounting for differences in strap use across functional ability levels or providing comparative data between groups. This lack of attention to functional differences in relation to restraint device use constitutes a significant gap. Understanding how strap use varies across different levels of functional ability is particularly important for improving athlete safety, optimizing performance, and supporting classification procedures. Addressing this gap, the present study aims to describe the use of different types of wheelchair straps in a population of professional WB players, further investigating the relationship between the use of straps and the players’ classification score. Considering the lack of literature on this topic, our initial hypothesis was null, as there were no prior references to support the expectation of specific outcomes. Nonetheless, we anticipated potential differences in strap use based on players’ classification scores.

## 2. Materials and Methods

### 2.1. Study Design

This study followed a cross-sectional design. An online survey was conducted using Google Forms, and completed questionnaires were collected via Google Drive. On 30 May 2024, the survey was electronically distributed to all WB players competing in the official Italian leagues organized by the Italian Wheelchair Basketball Federation (FIPIC). The survey was not piloted or validated beforehand, as it was specifically developed to address technical aspects of WB not covered by existing validated tools, thus requiring a tailored approach. The investigators sent the survey via direct email to a FIPIC representative, who then forwarded it to the team managers, who subsequently disseminated it to their players. Specifically, participant anonymity was ensured through a coding system implemented by FIPIC, which converted personal identifiers into alphanumeric codes prior to data transmission. As a result, the investigators received only de-identified datasets and had no access to information that could reveal the identity of the respondents. Furthermore, data were collected and analyzed in aggregate form, preventing the identification of individual participants. The study protocol was approved by the Institutional Review Board of the University of Salento (n.1/18 February 2024) and conducted according to the principles of the Declaration of Helsinki.

### 2.2. Participants

The sample consisted of professional WB players currently competing in the official Italian leagues organized by the FIPIC. All athletes included in the study had at least 24 months of experience in WB; therefore, any surveys completed by players who did not meet this requirement were excluded.

### 2.3. Survey Instruments

Every participant was asked to provide informed consent before completing the questionnaire. For participants under the age of 18, informed consent was obtained from their parents or legal guardians. The survey was developed following the design used in previous questionnaires of a cross-sectional study [15,26,27]. Sample questions from the questionnaire are available in the supplementary materials (Supplementary File S1). It included both open and closed questions, and it was divided into four sections:

- The first section provided information about the study and included the informed consent;
- The second section gathered demographic data and general information (e.g., sex, age, anthropometric characteristics, cause of motor disability, type of wheelchair use);
- The third section focused on the experience in WB (e.g., classification score verified via official records, playing role, years of experience in WB);
- The fourth and last section collected information about the use of wheelchair straps. Specifically, it included eight yes/no questions in which athletes indicated whether they use each specific type of strap (i.e., chest, abdominal, pelvic, thigh, leg, and foot strap).

### 2.4. Group Classification

The patient’s cohort was divided into two groups according to their classification score, as performed in other previous studies [18,28,29,30]: low-point players (LPPs) with scores between 1.0 and 2.5, and high-point players (HPPs) with scores between 3.0 and 4.5. This division is related to the IWBF Classification Manual, which refers to pelvic stability as a differentiator, with LPPs typically exhibiting passive pelvic stability, while HPPs generally demonstrate active pelvic stability [7].

### 2.5. Sample Size Calculation

The sample size for this study was determined through an a priori power analysis using G*Power (v3.1, Brunsbüttel, Germany). Expecting an effect size of 0.87 [31], considering α = 0.05 and power of 95%, we obtained a sample size of 35 subjects per group. Finally, assuming a 10% attrition rate, 39 subjects were considered for each group.

### 2.6. Statistical Analysis

All the statistical analyses were conducted in JASP (v0.19.3.0, University of Amsterdam). Continuous variables are presented as means ± standard deviation and range, while categorical variables are expressed as percentages of the total. The Chi-squared test was used to compare categorical variables between groups. A *p*-value < 0.05 was considered statistically significant.

## 3. Results

The sample size consisted of 82 WB players, whose demographic characteristics are described in Table 1. The sample included 43 participants in the LPP group and 39 in the HPP group.

Among the LPP participants, 4 out of 43 (9.3%) used a wheelchair exclusively for sports activities, while 39 out of 43 (90.7%) used a wheelchair in daily life. In contrast, within the HPP group, 23 out of 39 (58.97%) used a wheelchair only for sports activities, whereas 16 out of 39 (41.03%) used it in daily life (Table 2). Among the LPP participants, 32 out of 43 (74.42%) had spinal cord injuries as the cause of their disability, 9 (20.93%) had brain injuries, and 2 (4.65%) had limb injuries. In contrast, among the HPP participants, 23 out of 39 (58.97%) had limb injuries, 11 (28.21%) had spinal cord injuries, and 5 (12.82%) had brain injuries (Table 2).

Regarding classification score and the use of different types of straps, results are reported in Table 3.

A greater number of participants in the LPP group use chest and abdominal straps compared to those in the HPP group (20.93% vs. 5.13%), with a statistically significant difference (*p* = 0.036, χ^2^ = 4.397). The use of the pelvic strap does not differ significantly between the two groups (*p* = 0.921, χ^2^ = 0.010). The thigh strap is used by more participants in the HPP group than in the LPP group (97.44% vs. 81.40%), with a statistically significant difference (*p* = 0.020, χ^2^ = 5.385). Similarly, a higher proportion of HPP participants use the leg strap compared to those in the LPP group (43.59% vs. 23.26%) (*p* = 0.050, χ^2^ = 3.829). Conversely, the foot strap is used by more participants in the LPP group than in the HPP group (65.12% vs. 38.46%), with a statistically significant difference (*p* = 0.016, χ^2^ = 5.826).

## 4. Discussion

The present study aimed to describe the use of different types of wheelchair straps in a population of professional WB players. The results obtained from the survey showed significant differences in the use of various types of straps in relation to players’ classification scores.

### 4.1. Chest Strap

LPPs use the chest strap more than HPPs, with a statistically significant difference (*p* = 0.036). Previous research conducted by Curtis et al. investigated the influence of chest and thigh straps on trunk mobility in a population of WB players with classification scores ranging from 1.0 to 2.0 [32]. The study showed an increase in the area of functional reach in the sagittal plane when using a chest strap, compared to using a thigh strap or no strap at all. Although the chest strap led to greater improvements in the transverse plane in class 2.0 players compared to class 1.0 players, Curtis and colleagues highlighted that able-bodied control subjects did not gain any benefit in functional reach from the chest strap [32]. These findings are consistent with our results, considering that WB players with higher residual function tend to use the chest strap less than players with greater impairment. This may be attributed to the fact that chest straps offer more functional advantages to LPPs than to HPPs.

### 4.2. Abdominal Strap

The use of the abdominal strap follows a similar pattern to that of the chest strap, with a higher proportion of LPPs using it compared to HPPs (*p* = 0.036). This reflects the need for additional trunk support among players with greater impairment. Indeed, poor trunk control affects the pelvic tilt position, overloading the shoulders and putting these players at greater risk for overuse injuries [33,34]. Interestingly, abdominal straps are also commonly used in other Paralympic sports, such as wheelchair rugby, particularly among athletes with high spinal cord injuries. In these contexts, wheelchairs are often adjusted with a deep seating position and abdominal straps to compensate for reduced postural stability through alternative strategies [35]. Although abdominal straps offer benefits in terms of stability and performance, they can also limit trunk range of motion and force generation due to a reduced push angle [36].

In our study, both chest and abdominal straps were among the least commonly used by players with a classification score of 3.0 or higher, which aligns with their reduced need for external stabilization. These differences in equipment usage can be explained by the functional criteria of the WB classification system, particularly the distinction between passive and active pelvic stability. Players with a classification score of 2.5 or lower usually lack sufficient trunk and hip control to maintain a stable seated position while moving and, therefore, need external support, such as straps. In contrast, those with scores of 3.0 or higher generally demonstrate active pelvic stability and require minimal external support, as outlined in the IWBF Classification Manual [7]. This classification rationale aligns with higher chest and abdominal strap usage observed among LPPs in our study. Beyond classification, biomechanical factors such as the center of gravity also influence strap use. In fact, the center of gravity in individuals with paraplegia is positioned higher than in able-bodied individuals by an estimated three to four vertebral levels [37]. This upward shift leads to reduced postural stability, which is compounded by already compromised pelvic and trunk control due to paralysis of the associated musculature [32]. Furthermore, the higher the level of paraplegia, the greater the proportion of musculature without functional use, thereby increasing the need for external support or leading to overuse of the remaining innervated muscles. In summary, the present findings confirm that players with lower classification scores use chest and abdominal straps more frequently, in line with their greater need for trunk stabilization.

### 4.3. Pelvic Strap

Regarding pelvic strap, there are no statistically significant differences between the two groups, with a proportion of use ranging from 38.46% to 39.54%.

### 4.4. Thigh Strap

The findings of our research also highlighted that HPPs use the thigh strap more than LPPs, with a statistically significant difference (*p* = 0.020). The thigh strap has been previously studied in the scientific literature, particularly in comparison with the chest strap. Curtis et al. found that the thigh strap enables WB athletes with low point classification scores to slightly increase the area of their functional reach in the sagittal plane more than non-belted situation. However, the improvement was less than what could be reached with a chest strap [32]. Considering the transverse plane, as for the chest strap, class 2.0 players had greater benefit from the thigh strap compared to class 1.0 players. Thus, only wheelchair users with innervated trunk musculature who allow for trunk rotation can benefit from the use of a thigh strap. A thigh strap leads to pelvic stabilization but not to trunk stabilization [32], which is conversely more needed from athletes with less residual function. This could be a reason why LPPs use the thigh strap less than HPPs. While the thigh strap provides pelvic stabilization, it does not contribute to trunk stabilization [32], which is particularly important for athletes with limited residual function. This may explain why LPPs tend to use the thigh strap less than HPPs do.

### 4.5. Leg Strap

Concerning the leg strap, the results of the present study showed a statistically significant difference in its use, with HPPs using it more than LPPs do (*p* = 0.050). The scientific literature lacks studies specifically focused on this type of strap. As indicated in the IWBF Classification Manual, WB players with passive pelvic stability, and therefore typically with a low classification score, also require the use of a leg strap. Together with other wheelchair adjustments, such as an angled seat and additional straps, the leg strap helps them maintain an upright sitting position [7]. Moreover, in other adaptive sports, such as wheelchair tennis, leg straps are used to compensate for reduced pelvic stability. This condition is typical of athletes with a low classification score and is caused by a minimal or absent seat dump, often adopted to enhance players’ performance [20]. However, these statements are partially at odds with our findings, which highlight a greater use of the leg strap among HPPs compared to LPPs. Considering that the majority of HPP participants in our sample had limb injuries—most likely lower limb amputations—we hypothesize that this higher use of the leg strap may be related to the need to secure the stump to the wheelchair. This would allow the athlete to move in complete harmony with the wheelchair. This interpretation is speculative and should be further explored in future research. However, it is partially supported by the IWBF Official Interpretations [23], which clarify that players with double leg amputations may secure their legs to the wheelchair below the knees only if this is indicated on their classification card. Such securing, whether by strapping, prosthesis, or wheelchair design, may provide a considerable advantage. In fact, a below-knee amputee with a secured stump is considered functionally equivalent to having a complete limb for classification purposes. This regulatory perspective reinforces the strategic use of leg straps among HPPs, not only for stability but also to optimize postural anchoring and kinetic synergy with the wheelchair, thereby enhancing control and maneuverability.

### 4.6. Foot Strap

Another statistically significant difference identified in our study concerns the use of the foot strap. It emerged that LPP athletes used the foot strap more frequently than HPP athletes (*p* = 0.016). This type of strap is used to secure the athlete firmly to the seat, allowing the athlete and wheelchair to act in concert and granting higher stability [38,39]. Similarly to the leg strap, the IWBF Classification Manual indicates that WB players with passive pelvic stability particularly require the use of a foot strap to sustain a right seated posture [7]. Furthermore, we can speculate that the lower use of the foot strap among HPP athletes may be related to the higher prevalence of individuals with lower limb lesions within this group.

These findings may provide useful insights for wheelchair configuration during practices and matches, injury prevention, and classification procedures in WB. Coaches and team staff could use information about the use of straps to guide individualized wheelchair configuration, improve postural stability and movement efficiency while minimizing the risk of injuries, particularly those related to falls. Physiotherapists may also benefit from a better understanding of strap use to support trunk control and minimize potential musculoskeletal overload, given their significant role in physical assessment and injury management. For classifiers, athletes’ preferences regarding restraint devices may provide additional cues to refine functional assessments and ensure greater alignment between classification scores and athletes’ actual support needs. Furthermore, combining information about strap use with other variables of setup underscores the importance of a holistic approach to equipment customization. However, while our findings mainly reflect functional considerations related to classification scores, it is also important to acknowledge other factors. These may include athletes‘ personal preferences, coaching habits and instructions, or regional equipment rules, which may also influence strap use patterns.

This study is not free of limitations. Firstly, data were obtained through an online survey, which may have resulted in occasional inaccuracies in self-reported information. Nevertheless, this approach enabled the recruitment of a large cohort of players, thereby ensuring greater sample representativeness. With regard to the survey’s design, it did not include previously validated questionnaires, as it was specifically developed to investigate aspects of WB that are not currently addressed by any existing validated tools in the literature. Secondly, another limitation of this study concerns potential selection and response bias due to voluntary participation in the online survey. Moreover, the findings may not be generalizable to all populations or settings due to specific sample characteristics, as the sample was limited to WB players competing in the official Italian leagues, including foreign players competing in Italy. Furthermore, a potential limitation is the use of the classification score as a proxy for functional capability. Although it may not fully represent the complexity of an athlete’s functional capability, it remains an objective and standardized parameter, widely adopted in WB competitions and frequently used as a reference point in the scientific literature. Finally, the limited availability of previous research specifically related to this topic required a more interpretative approach to data analysis and discussion. It also limited the possibility of exploring alternative evidence-based explanations involving other influencing factors. Consequently, some considerations are based on reasoned assumptions, which should be taken into account when interpreting the present findings, while also offering a valuable starting point for future investigations.

However, to our knowledge, this research is the first focused on the use of wheelchair straps and their relationship with classification score, thus determining the main strength of this article. Moreover, it collects a significant amount of data relating to elite athletes practicing WB; therefore, it constitutes a reliable source of scientific data relevant to this area of research within the Paralympics. Future studies are required to further investigate the role of restraint devices in WB, especially with larger samples to enhance generalizability. In particular, biomechanical validation studies and longitudinal research are needed to better understand the functional impact of strap use over time and its relationship with additional functional parameters.

## 5. Conclusions

The present study investigated the use of different types of wheelchair straps in a population of professional WB players. The results demonstrated significant differences in the use of various types of straps in relation to players’ classification scores. Players with lower classification scores tended to use chest, abdominal, and foot straps more frequently. In contrast, thigh and leg straps were more commonly used by players with higher classification scores. Given the limited literature on this topic, further studies are needed to deepen these results and to enrich the knowledge about restraint devices, particularly in light of their importance in improving performance and balance of WB players.

## Figures and Tables

**Table 1:** Sample demographic characteristics.

Variable	Value
Age, mean ± SD (range), y	28.96 ± 9.62 (16–49)
Male, n (%)	75 (90.4)
Height, mean ± SD (range), cm	171.55 ± 14.39 (130–198)
Weight, mean ± SD (range), kg	65.38 ± 13.6 (33.6–96)
Cause of disability, n (%)	
Spinal cord injuries	43 (51.8)
Brain injuries	14 (16.9)
Limb injuries	26 (31.3)
Use of wheelchair, n (%)	
Limited to sports practice	28 (33.7)
Habitual in daily activities	55 (66.3)
Classification score, n (%)	
LPP	43 (52.4)
HPP	39 (47.6)
Years of experience in WB, mean ± SD (range), y	10.34 ± 6.84 (1–28)

Demographic characteristics of the study sample. Values are reported as mean ± standard deviation (SD) with range, or as number (n) and percentage (%), as appropriate. Abbreviations: SD, standard deviation; y, years; n, numbers; cm, centimeters; kg, kilograms; LPPs, low point players; HPPs, high point players; WB = wheelchair basketball.

**Table 2:** Wheelchair use and cause of disability by classification score.

	Classification Score		
	LPP	HPP	*p*-Value
Use of wheelchair, n (%)			
Limited to sports practice	4 (9.30)	23 (58.97)	**<0.001**
Habitual in daily activities	39 (90.7)	16 (41.03)	**<0.001**
Cause of disability, n (%)			
Spinal cord injuries	32 (74.42)	11 (28.21)	**<0.001**
Brain injuries	9 (20.93)	5 (12.82)	**<0.001**
Limb injuries	2 (4.65)	23 (58.97)	**<0.001**

Distribution of wheelchair use and cause of disability among players, stratified by classification score. Abbreviations: LPPs, low point players; HPPs, high point players; n, numbers.

**Table 3:** Use of the strap in relation to the classification score.

	Classification Score		
	LPP	HPP	*p*-Value
Use of chest strap, n (%) Yes No	9 (20.93) 34 (79.07)	2 (5.13) 37 (94.87)	**0.036**
Use of abdominal strap, n (%) Yes No	9 (20.93) 34 (79.07)	2 (5.13) 37 (94.87)	**0.036**
Use of pelvic strap, n (%) Yes No	17 (39.54) 26 (60.46)	15 (38.46) 24 (61.54)	0.921
Use of thigh strap, n (%) Yes No	35 (81.40) 8 (18.60)	38 (97.44) 1 (2.56)	**0.020**
Use of leg strap, n (%) Yes No	10 (23.26) 33 (76.74)	17 (43.59) 22 (56.41)	**0.050**
Use of foot strap, n (%) Yes No	28 (65.12) 15 (34.88)	15 (38.46) 24 (61.54)	**0.016**

Distribution of different types of wheelchair straps, stratified by classification score. Abbreviations: LPPs, low point players; HPPs, high point players; n, numbers.

## Data Availability

The data presented in this study are available on request from the corresponding author. The data are not publicly available due to privacy and ethical restrictions.

## Supplementary Materials

The following supporting information can be downloaded at: https://www.mdpi.com/article/10.3390/sports13070222/s1, Supplementary File S1: Sample questions from the questionnaire used in this study (DOCX).

## Abbreviations

The following abbreviations are used in this manuscript:

WB	Wheelchair basketball
IWBF	International Wheelchair Basketball Federation
LPPs	Low-point players
HPPs	High-point players
