# Supplementary material for: The Relationship Between Strap Use and Classification Score in Elite Wheelchair Basketball Players
Source: Sports (Basel). 2025 Jul 8;13(7):222. doi: 10.3390/sports13070222 (PMC12297868; doi:10.3390/sports13070222)
Supplement: Supplementary file 1 [file sports-13-00222-s001.zip › sports-3681242-supplementary.pdf]

Supplementary Material

Examples of Survey Questions

Please indicate your height in centimeters (approximate is acceptable) (e.g., 176):

____

Please indicate the cause of your motor disability:

- ○ Spinal cord injury
- ○ Brain injury
- ○ Limb injury

Please indicate your use of the wheelchair:

- ○ Limited to sport practice
- ○ Habitual in daily activities

Please indicate which type(s) of strap you use when playing wheelchair basketball:

- ○ Chest strap
- ○ Abdominal strap
- ○ Pelvic strap
- ○ Thigh strap
- ○ Leg strap
- ○ Foot strap
